# Impact of STING Inflammatory Signaling during Intracellular Bacterial Infections

**DOI:** 10.3390/cells11010074

**Published:** 2021-12-28

**Authors:** Erika S. Guimarães, Fabio V. Marinho, Nina M. G. P. de Queiroz, Maísa M. Antunes, Sergio C. Oliveira

**Affiliations:** 1 Departamento de Genética, Ecologia e Evolução, Instituto de Ciências Biológicas, Universidade Federal de Minas Gerais, Belo Horizonte 31270-901, Brazil; erikasousaguimaraes@gmail.com; 2 Departamento de Bioquímica e Imunologia, Instituto de Ciências Biológicas, Universidade Federal de Minas Gerais, Belo Horizonte 31270-901, Brazil; fabiovitarelli@yahoo.com.br (F.V.M.); ninagual@gmail.com (N.M.G.P.d.Q.); maisaantunes@gmail.com (M.M.A.); 3 Instituto Nacional de Ciência e Tecnologia em Doenças Tropicais (INCT-DT), CNPq MCT, Salvador 40110-160, Brazil

**Keywords:** STING, bacteria, type I interferon, infection, inflammation, cyclic dinucleotides

## Abstract

The early detection of bacterial pathogens through immune sensors is an essential step in innate immunity. STING (Stimulator of Interferon Genes) has emerged as a key mediator of inflammation in the setting of infection by connecting pathogen cytosolic recognition with immune responses. STING detects bacteria by directly recognizing cyclic dinucleotides or indirectly by bacterial genomic DNA sensing through the cyclic GMP-AMP synthase (cGAS). Upon activation, STING triggers a plethora of powerful signaling pathways, including the production of type I interferons and proinflammatory cytokines. STING activation has also been associated with the induction of endoplasmic reticulum (ER) stress and the associated inflammatory responses. Recent reports indicate that STING-dependent pathways participate in the metabolic reprogramming of macrophages and contribute to the establishment and maintenance of a robust inflammatory profile. The induction of this inflammatory state is typically antimicrobial and related to pathogen clearance. However, depending on the infection, STING-mediated immune responses can be detrimental to the host, facilitating bacterial survival, indicating an intricate balance between immune signaling and inflammation during bacterial infections. In this paper, we review recent insights regarding the role of STING in inducing an inflammatory profile upon intracellular bacterial entry in host cells and discuss the impact of STING signaling on the outcome of infection. Unraveling the STING-mediated inflammatory responses can enable a better understanding of the pathogenesis of certain bacterial diseases and reveal the potential of new antimicrobial therapy.

## 1. Introduction

The first line of defense against invasive microbial agents involves various families of germ-line encoded pattern recognition receptors that recognize specific pathogen-associated molecular patterns. In this sense, cytosolic recognition and host defense against foreign genetic material are pivotal features conserved in different species across 600 million years of evolution [1,2]. The endoplasmic reticulum (ER)-associated protein stimulator of interferon genes (STING) encompasses a vital part of this immune cytosolic surveillance system. STING acts as a direct sensor in the recognition of cyclic dinucleotides (CDNs) originating from intracellular bacteria or as an adaptor, detecting DNA indirectly, through cyclic GMP-AMP synthase (cGAS) [3].

STING activation culminates in a robust inflammatory response associated with interferon regulatory factor 3 (IRF3) and the subsequent transcription of type I interferon (IFN) and other co-regulated genes. Additionally, STING signaling mediates nuclear factor kappa B (NF-kB) activation and proinflammatory cytokine secretion [3]. Cytokine production is essential for inducing an effective innate host defense. In this context, type I IFN boosts cell autonomous defense mechanisms and is associated with efficient immune responses. However, the role of type I IFN during bacterial infections is still controversial, and resulting outcomes can either be protective or deleterious to the host, depending on the infection [4].

In addition to sensing bacteria directly, the canonical relationship with cGAS broadens STING potential in participating in immune responses, and its signaling has been associated with inflammatory, autoimmune, and infectious diseases [5]. The role of STING in some bacterial infections was previously compilated [6]. In this review, we expanded the knowledge of STING function for other bacterial pathogens and, we addressed here the emerging features of STING activation, such as the recently reported relationship with macrophage polarization, immunometabolism, and ER stress, setting STING as a major driver of inflammatory responses [7]. This review addresses these novel breakthroughs concerning the multifaceted role of STING as a regulator of inflammatory immune responses, with a particular focus on intracellular bacterial infections.

## 2. Basis of STING Activation

In different situations, components, such as lipopolysaccharide, nucleic acids, and CDNs, can be released within the cytosol. In this scenario, the mechanisms of STING activation mainly include the binding to bacterial CDNs, such as cyclic-di-GMP, the first identified ligand for STING, and cyclic-di-AMP generated by Gram-positive bacteria, such as *Listeria* [8,9]. CDNs are important second messenger signaling molecules, broadly distributed throughout prokaryotes that orchestrate multiple physiologic processes, including virulence, motility, and biofilm formation [10]. Upon binding to DNA, cGAS generates the “non-canonical” CDN cyclic GMP-AMP (cGAMP) that serves in this context as the bona fide ligand for STING [11,12]. Remarkably, the binding of CDNs is an evolutionary ancient STING function in animals [1], indicating that STING initially evolved as a CDN sensor and subsequently co-opted for DNA sensing. Interestingly, human STING variants evolved to distinguish the canonical CDNs produced by bacteria from the CDN produced by cGAS [12].

In addition to bacterial infections, the cGAS–STING axis can recognize DNA from numerous sources, including viral DNA, released mitochondrial DNA, extranuclear chromatin, and cytosolic micronuclei. In this context, cGAS is required for responding to DNA virus infection and cGAS deficient mice are more susceptible to infection with numerous DNA viruses, including herpes simplex cytomegalovirus and vaccinia virus [13]. This signaling pathway also plays an important role in RNA virus control, including vesicular stomatitis virus, dengue, and West Nile virus [14], but immune responses are, at least, partially associated with the indirect detection of mitochondrial DNA [15]. Retroviruses, such as the human immunodeficiency virus (HIV), can also activate the cGAS–STING pathway [16]. Further, the cGAS–STING axis is also associated with the detection of parasites, such as *Plasmodium* [17] and *Schistosoma mansoni* [18]. In addition to infection-associated DNA, cGAS can also detect endogenous self-DNA that gains access to the cytosol in the context of cell damage or stress. This includes conditions associated with increased cell death, mitotic stress, cellular senescence, defective mitosis, and DNA and mitochondrial damage [19]. Enhanced cytosolic DNA is related to, for instance, sterile injuries, cellular senescence, cancer, and autoimmune diseases [19].

On a structural level, cGAS binds to cytosolic DNA from these various sources in a length-dependent manner. Short dsDNA (smaller than 20 bp) can bind to cGAS, but longer DNA (larger than 45 bp) induce stronger enzymatic activity [20]. Interestingly, cGAS positioned at the plasma membrane prevents recognition of self-DNA and enables the proper sensing of viral infections, ensuring self-nonself discrimination [21]. Although other sensors, including the dead box helicase 41 (DDX41) and interferon inducible protein 16 (IFI16), also detect cytosolic DNA, cGAS is considered the main sensor required for DNA-mediated activation of STING [22,23].

In its inactive state, STING resides in the ER membrane as a constitutive dimer composed of a four-span transmembrane domain, a connector region, a cytosolic N-terminal segment, and a cytosolic ligand-binding domain (LBD), to which a C-terminal tail (CTT) is attached [24,25]. Upon activation, STING undergoes extensive conformational changes, including untwisting (180° rotation) of the LBD, which forms a ligand-binding pocket and allows STING oligomerization and lateral stacking. The oligomerized STING dimers form the activated STING unit capable of triggering effector functions [26]. STING dimers then associate, via their CTT, with TANK-binding kinase 1 (TBK1). A prerequisite for TBK1 recruitment and downstream signaling is the STING transit from the ER to the Golgi, through the ER–Golgi intermediate compartments (ERGIC), in a process dependent on the coatomer protein complex II (COPII) vesicles [27]. Then, TBK1 auto-phosphorylates and phosphorylates STING’s CTT, forming a docking site that recruits IRF3, which is subsequently phosphorylated by nearby TBK1. Following IRF3 activation, the STING–TBK1–IRF3 complex is dissociated, and activated IRF3 dimerize and translocate to the nucleus to induce type I IFNs and interferon-stimulated genes (ISGs) [28,29]. In addition, STING also modulates NF-κB activation, independently on the CTT. Although TBK1 itself can promote NF-κB activation, in some contexts TBK1 is not fully required for NF-κB activation downstream of STING [30]. NF-κB synergizes with IRF3 and prompts high levels of type I IFNs, and induces the transcription of other proinflammatory cytokines, such as interleukin (IL)-6 and tumor necrosis alpha (TNF-α) [31]. Alternatively, STING trafficking also induces noncanonical autophagy that degrades DNA and bacteria from the cytosol, apoptosis, and necroptosis [32,33].

Type I IFNs bind a transmembrane IFN receptor (IFNAR), which is composed of two subunits (IFNAR1 and IFNAR2). In the canonical type I IFN-induced signaling pathway, IFNAR engagement leads to Janus kinase 1 (JNK1) and tyrosine kinase 2 (TYK2) activation, which further phosphorylate the signal transducer and activator of transcription 1 (STAT1) and 2 (STAT2). Tyrosine-phosphorylated STAT1 and STAT2 dimerize and migrate to the nucleus, activating interferon regulatory factor 9 (IRF9) to form IFN-stimulated gene factor 3 (ISGF3), a trimolecular complex. ISGF3 binds to its specific DNA sequences, which are known as IFN-stimulated response elements that can activate ISGs transcription [34,35]. Finally, ISG-encoded proteins can influence pathogen spread due to several different intracellular mechanisms.

STING activation is an immunologic event not only related to host defense against pathogens, but also during sterile injuries [36]. Therefore, strategies developed to modulate STING activation in immune cells emerge as a promising venue for investigation in various diseases.

## 3. STING and Endoplasmic Reticulum Stress

STING localization in the ER supports its response to cellular and organelle stress. The ER is a multifaceted organelle that orchestrates diverse physiological processes, including protein folding and translocation. Under certain physiological and pathological conditions—including increased protein demand, nutrient deprivation, hypoxia, and calcium dysregulation—the abundance of unfolded proteins exceeds the ER folding capacity, triggering ER stress [37]. The unfolded protein response (UPR) is a highly conserved adaptative signal transduction pathway, triggered to counteract elevated ER stress and restore cell proteostasis. In response to ER stress, the UPR coordinates a plethora of responses that ultimately increase the ER capacity through the augmentation of ER folding, regulation of the ER-associated protein degradation (ERAD) pathway, and selective mRNA degradation repression [38,39]. The UPR is governed through the activation of three primary ER membrane resident stress sensors, inositol-requiring enzyme 1 (IRE1), protein-kinase r-like endoplasmic reticulum kinase (PERK), and activating transcription factor 6 (ATF6) (reviewed in detail in Hetz et al. [40]). Together these pathways re-establish proteostasis by decreasing the protein load and enhancing the ER folding capacity. Nonetheless, upon persistent UPR activation, these pathways can lead to apoptotic cell death [41].

ER stress is emerging as a driver of several disorders, as ER stress intersects and activates innate immunity and pro-inflammatory signaling [42,43]. In this scenario, increasing evidence supports the crosstalk between STING and the UPR. This strong interplay between STING and ER stress was demonstrated in several non-pathogenic diseases, including alcoholic liver disease and liver fibrosis [44,45], and also during bacterial infections [46]. In this regard, an intricated link between ER stress, STING, and apoptosis was demonstrated during *Mycobacterium bovis* infection. Treatment with 4-Phenylbutyric acid (4-PBA (an ER stress-inhibitor)) reduced the phosphorylation of TBK1 and IRF3 and cytoplasmatic co-localization of STING and TBK1. *M. bovis* infection led to the interaction of fully activated IRF3 with the apoptosis regulator Bax, culminating in mitochondrial damage and apoptosis. It is worth noting that intracellular bacterial survival increased upon ER-stress and IRF3 blocking [47]. During *Listeria innocua* infection, detection of the bacterial second messenger ci-di-AMP through STING was described as a trigger for ER stress. Subsequent inactivation of the mammalian target of rapamycin (mTOR), induced the canonical autophagy that sequestered stressed ER membranes, contributing to ER-stress resolution. This ER-phagy favored the survival of phagocytes, and localized STING to autophagosomal membranes as a prelude to the production of type I IFNs [48]. Moreover, our group recently demonstrated that *Brucella abortus* triggers the UPR in a STING-dependent manner, also dependently on the recognition of bacterial CDNs. This *Brucella*-induced UPR was crucial for triggering several proinflammatory responses. Interestingly, UPR was induced in a 2 waves manner during a 24 h period, suggesting that it can influence, per se, the production of type I IFNs, guanylate binding proteins (GBPs), IL-1β, and IL-6 in latter moments during infection. Notwithstanding, UPR induced by *Brucella* was detrimental for the host [49].

## 4. Macrophage Polarization: STING Activation as an Inflammatory Inducer

Macrophages metabolic reprogramming in response to microbial insults can directly influence the outcome of infection [50]. Recently, several reports have linked macrophage reprogramming and immunometabolism to STING activation during infections, which is the focus of the following discussion.

The most recent understanding related to the classification of macrophages in only two groups—classic activated and alternative activated—has been replaced by the concept that this classification is more complex, as there is a broad polarization spectrum that can change the macrophage profile, depending on the source of macrophages, activators, and a collection of markers to describe macrophage activation [51]. Macrophages polarized to an inflammatory profile (M1) are characterized by the production of inflammatory cytokines, such as TNF-α, IL-6, and IL-1β, nitric oxide (NO) production, and glycolytic metabolism. On the opposite side of the spectrum, we have anti-inflammatory macrophages (M2), characterized by anti-inflammatory cytokines (e.g., IL-10), enhanced STAT6 activation, and enhanced mitochondrial oxidative phosphorylation (OXPHOS) activity, leading to an immunosuppressive response and tissue remodeling [51,52]. In this regard, STING has been associated with macrophage reprogramming. For example, STING modulated the severity of intestinal inflammation in experimental colitis in mice through macrophage polarization. In this case, STING agonists, including c-GAMP, cyclic-di-AMP, and the murine agonist 5,6-Dimethylxanthenone-4-acetic acid (DMXAA), induced the repolarization of anti-inflammatory macrophages towards an inflammatory phenotype, as evidenced by decreased *Arg1* and *Fizz1* expression and the induction of *Nos2* and IL-12p40. Importantly, these effects were greatly dampened in *TMEM173^gt^* (STING-mutant) macrophages [53,54].

**Figure 1:**
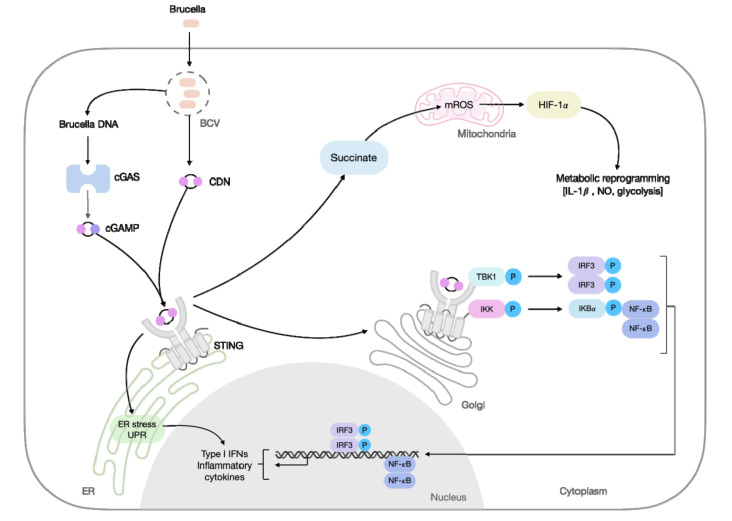
STING signaling in response to *B. abortus* infection. The intracellular bacteria, *B. abortus*, enters the host cell and forms the Brucella containing vacuole (BCV), to ensure survival. Upon the guanylate binding proteins (GBP)-mediated lysis of the BCV, bacterial components, such as Brucella DNA and bacterial cyclic di-nucleotides (CDNs), are exposed in the cytosol. The stimulator of interferon genes (STING) can directly sense bacterial CDNs or indirectly sense the Brucella DNA through the cyclic GMP-AMP synthase (cGAS). The recognition of CDNs through STING, triggers endoplasmic reticulum (ER) stress and the unfolded protein response (UPR), culminating in type I interferon (IFN) and inflammatory cytokine production, through pathways not completely understood. Additionally, activated STING traffics from the ER to the Golgi, which facilitates TANK-binding kinase 1 (TBK1) recruitment and phosphorylation. Activated TBK1, phosphorylates interferon regulatory factor 3 (IRF3) and nuclear factor kappa B (NF-κB) to induce type I IFNs and other inflammatory cytokines. Additionally, STING contributes to the metabolic reprogramming in macrophages. STING activation, during *B. abortus* infection, leads to the accumulation of the metabolite succinate, through pathways not completely understood, which in turn favors mitochondrial ROS (mROS) generation. Succinate and mROS drive hypoxia-inducible factor1-alpha (HIF-1α) stabilization, leading to enhanced IL-1β release, nitric oxide (NO) production, and induction of a glycolytic metabolic profile. cGAMP: cyclic GMP-AMP; IKK: IκB kinase complex; and IκBα: nuclear factor of kappa light polypeptide gene enhancer in B-cells inhibitor alpha.

STING polarization features have also been increasingly related to bacterial infections. STING signaling plays a key role during *Brucella abortus* infection [49,55]. Recently, our group demonstrated that STING is involved in the metabolic reprogramming of macrophages during *B. abortus* infection, by polarizing macrophages towards an inflammatory profile characterized by the enhanced expression of M1 markers (e.g., C-C chemokine receptor type 7 (Ccr7), Nos2, and CD80), and down-regulated the expression of anti-inflammatory macrophage-related markers (e.g., Arg1, chitinase-like 3 (Ym1), CD163, and CD206) [56]. Mechanistically, this metabolic reprogramming was induced by STING-dependent stabilization of hypoxia-inducible factor-1 alpha (HIF-1α), and HIF-1α signaling enhanced glycolytic metabolism and diminished OXPHOS upon infection. STING drives HIF-1α stabilization through increased succinate and mitochondrial ROS (mROS), leading to an enhanced NO production, inflammasome activation, and IL-1β release. Notably, the increased presence of proinflammatory macrophages helps restrain the *B. abortus* infection (Figure 1) [56]. During *Mycobacterium tuberculosis (Mtb)* infection, STING, and downstream type I IFN, restrains macrophage metabolism. IFN-β itself, rather than direct bacterial factors, prevented the shift to aerobic glycolysis and induced mitochondrial damage in inflammatory macrophages. Interestingly, STING signaling acted upstream of the mitochondrial damage [57]. Furthermore, Benmerzoug et al. demonstrated that the mechanism underlying the impaired control of *Mtb* observed during lung inflammation induced by silica pre-treatment, correlated with induction of M2 macrophages. Silicosis decreased *Nos2* expression and enhanced *Mrc1* and *Arg1* expression in *Mtb*-infected mice dependent on STING. In this scenario, the self-DNA released upon silica-induced lung damage was the key molecule associated with STING priming, which potentiated *Mtb* sensing and mediated M2-macrophage polarization, crucial for the impaired control of host infection [58]. Further, the relevance of STING for the polarization of inflammatory macrophages has stimulated studies with STING agonists, such as CDNs formulated in a protein subunit vaccine against *Mtb* in the mouse model [59]. Another strategy was demonstrated using a recombinant BCG (BCG-*disA*-OE), which overexpresses c-di-AMP, inducing a robust M1 phenotype with increased levels of TNF-α, IL-1β, and IL-6, and an enhanced protection against pulmonary tuberculosis (TB) [60] and bladder cancer [61]. Targeting STING to induce an effective immune response can contribute to the development of future therapies.

## 5. STING Role during Intracellular Bacterial Infections

The features of STING activation are notoriously increasing. Since the emerging roles of this pathway during bacterial infections were previously compilated [6], new players were added to the scene and unanticipated mechanisms were revealed. For the first time, the information of STING relevance on *Legionella pneumophila*, *Yersinia pestis*, and *Burkholderia* spp. infection was gathered. On the other hand, the investigation on STING-dependent mechanisms, resulting from host interaction, with the previously addressed genera *Chlamydia*, *Listeria*, *Salmonella*, *Francisella*, *Brucella*, and *Mycobacterium* has extended, and these newly addressed responses are discussed in the present paper. The next section highlights these recent breakthroughs, focusing on STING-induced immune responses against intracellular bacteria.

*Legionella pneumophila* causes the pneumonia commonly referred to as Legionnaire’s disease. It possesses a type IV secretion system (T4SS) that is responsible for the assembly of a specialized replication vacuole, which recruits secretory vesicles from the ER, mitochondria, and ribosomes [62]. The IFN-β production in this context is dependent on the presence of functional T4SS and STING [62]. Despite the c-di-GMP in *L. pneumophila* being able to induce type I IFN production [63], the bacterium DNA is the main STING pathway activator [62,64]. Cells from individuals harboring *R232H TMEM173* polymorphism that impairs activation driven only by bacterial CDNs, had a fully functional immune response against this bacterium. However, cells with *HAQ TMEM173* polymorphism, which hampers the mediated responses of both DNA and bacterial CDNs, presented reduced IFN-β and proinflammatory cytokines production in response to *L. pneumophila* [64]. Additionally, a positive association between haplotype HAQ carriers and increased susceptibility to Legionnaire’s disease in European case-control cohorts was observed [64]. It is interesting to note that the absence of IFNAR signaling in mice does not increase their susceptibility to infection, whereas the absence of cGAS or STING slightly does increase their susceptibility to infection [62,64]. Moreover, it was suggested that extracellular vesicles from *L. pneumophila*-infected cells can mediate STING activation in bystander cells, a feature that needs further investigation [65]. These studies place STING as an important sensor for *Legionella* infections and indicate that other functions linked to this sensor, in addition to type I IFN response, are important for bacterial control.

The pathogen –STING relationship was recently investigated in *Yersinia pestis* infection. This Gram-negative bacterium possesses a T3SS responsible for the injection of effector proteins that inhibit immune signaling. The protein YopJ gains access to the cytoplasm through this mechanism, and has the peculiar ability to deubiquitinate STING, blocking its association with TBK1 and further IRF activation [66]. Although YopJ can inhibit other intracellular signaling pathways, the silencing of bacterial DNA recognition itself is sufficient for promoting *Y. pestis* infection, resulting in increased pathological severity and bacterial burden [66].

An unexpected form of STING activation was observed in *Burkholderia* spp. infection. This pathogen is the causative agent of melioidosis, a disease with a wide spectrum of clinical manifestations. *Burkholderia* has a peculiar intracellular life cycle that involves the fusion of infected cells with their neighboring healthy cells. This process is dependent on its T6SS5, resulting in the formation of multinucleated giant cells [67]. Ku et al. showed that the cell fusion induces cGAS–STING activation, due to the formation of micronuclei, as a result of abortive division. The DNA damage generated in this process is the triggering signal. Subsequently, there is a STING-dependent activation of autophagy, ultimately leading to cell death and the release of bacteria. Although, in this context, there is *IFNB1* expression, IFN-β is produced in low concentrations and the IFNAR signaling was not relevant for the cytotoxicity. Despite this intricate mechanism of host–pathogen interaction, the cGAS–STING pathway is not relevant to alter in vivo infection outcomes [67].

The obligatory intracellular pathogen, *Chlamydia* spp., presents a biphasic life cycle comprising the replicative reticulate bodies and the infectious elementary bodies. Both stages can activate the STING pathway [55]. Recently, it was shown that type I IFN production, resulting from this signaling, contributed to inflammasome activation [68]. The authors showed that targeting the STING/interferon pathway, can provide useful vaccine adjuvant and therapeutic targets to aid the treatment of *Chlamydia trachomatis* infection and its associated inflammatory pathology. In addition to this, STING can mediate cell death as a protective response during chlamydial infection [69]. The intricate relationship between *Chlamydiae* and the STING pathway was reviewed by Wen and Li [70], and they suggested that STING is protective against infection. However, in order to clearly define how STING-mediated responses affect the pathogenesis of *Chlamydia* infections, additional in vivo studies are needed.

*Listeria monocytogenes* activates STING, by both its genomic DNA and c-di-AMP production, resulting in a marked type I IFN production during infection [6]. Nandakumar et al. showed that extracellular vesicles (EVs) from *L. monocytogenes* infected cells are another form of cGAS–STING pathway activation. The bacterial DNA is packed in exosomes, in a mechanism dependent on MVB12, a protein that influences sorting and cargo in multivesicular bodies. These EVs are then transferred to bystander cells, in which the DNA will activate cGAS–STING and downstream signaling. Interestingly, this process is induced by STING activation in the donor cell [65]. This mechanism was also evoked in *Francisella tularensis* infected cells [65]. During systemic in vivo infection, the *L. monocytogenes* infected cell-derived EVs promote T cell apoptosis, resulting in decreased bacterial clearance [65]. Conversely, STING activation helps bacterial killing in intestines, in orally-induced *L. monocytogenes* enterocolitis. The investigators found that STING activation led to reduced bacterial burden and correlated with the recruitment of monocytes to the intestines during *L. monocytogenes*-induced enterocolitis. This STING-mediated protective response was triggered by the secretion of *L. monocytogenes* c-di-AMP, while the disruption of type I IFN signaling during *L. monocytogenes*-induced enterocolitis did not recapitulate STING deficiency [71]. Strikingly, IFNAR and IRF3/7 signaling were detrimental for the host protection against *Listeria*-induced enterocolitis [71]. These results suggest that STING activation can be crucial for infection resistance in specific tissues, and that in some situations its antibacterial properties are unrelated to type I IFN production. A similar approach of orally-induced bacterial enterocolitis was addressed to investigate STING relevance on *Salmonella typhimurium* infection. In this model, Th17 polarization was partially dependent on STING activation [72]. However, the T cell polarization and resistance to infection were greatly dependent on IRF1 [72], corroborating previous findings that TLR signaling can be the major pathway responsible for *Salmonella*’s induced immune response [6].

New features of STING activation were discovered as influencing *Brucella* spp. infection. It is now confirmed that both bacterial-derived DNA or c-di-GMP can induce STING activation [49,55]. Moreover, an intricate host–pathogen relationship was discovered as governing STING signaling. As noticed with other bacterial infections, STING activation partially influenced proinflammatory cytokine production, but was important for type I IFN expression [49,55]. Next, distinct paths can be tracked. First, in response to STING-induced type I IFN signaling, GBPs are induced, resulting in the rupture of *Brucella*-containing vacuoles and the release of the bacterium and its contents into the cytosol. Then, bacterial DNA activates AIM2 inflammasome, culminating in IL-1β production and control of infection both in vitro and in vivo [55]. It is worth noting that the STING-mediated intracellular release of *Brucella* components can have consequences in other intracellular pathways, such as non-canonical inflammasome activation via caspase-11 [73] and increased inflammatory macrophages that help restrain *Brucella* infection [56]. Such a scenario is supported by the finding of a higher bacterial burden, associated with an increased presence of IL-4 producing T lymphocytes and alternatively activated macrophages in mice deficient of STING [55,56]. However, in the attempt to survive the battle, *Brucella* species try to subvert the immune recognition by suppressing STING activation. This feature was shared by all tested *Brucella*, namely *B. abortus*, *B. melitensis*, *B. neotomae*, and *B. suis*. The infection by *Brucella* leads to the downregulation of STING over time, through the induction of microRNA miR-24, in a manner dependent on its T4SS and viable bacteria. Although miR-24 can downregulate other proteins, in vitro analysis suggested that STING is the major target in macrophages, since the absence of the locus *miR23a*, which is responsible for miR-24 expression, renders mice resistant to infection [74]. This suggests that a common strategy of the *Brucella* species is to induce miR-24 expression, to overcome STING and increase infection success. Thus, STING is a valuable sensor for the host to rely on during the fight against *Brucella* infection, and it is able to induce direct and indirect immune pathways that, so far, mainly account for a protective outcome.

The genus *Mycobacterium* comprises a diverse set of pathogens. The studies concerning STING relevance on these infections are diverse, as well. Genomic bacterial DNA seems to be the main activator of STING via cGAS [6]. Nevertheless, more investigations on STING–*Mycobacterium* relationships continue to emerge. In this regard, it was shown that this sensor participates in the activation of DCs in response to *Mtb* and *M. bovis* infection, influencing the production of cytokines (type I IFNs among them) and the expression of activation markers [75,76]. Additionally, inflammasome induction during mycobacterial infection can negatively regulate STING. The adaptor molecule ASC, through its caspase recruitment domain (CARD), interacts with the STING C terminus domain, impeding TBK1 association and, consequently, type I IFNs expression [77]. Moreover, it was discovered that the *Mtb* secreted protein, MmsA, whose functions range from bacterial metabolism to immune activation, promotes STING degradation by autophagy, repressing type I IFN production [78]. The ASC- and MmsA-induced mechanisms of STING downregulation are associated, respectively, with the TB type I IFN signature and *Mtb* strain hypervirulence [77,78]. However, these in vitro relationships are not translated into a leading character in vivo as, during *Mtb* infection, STING is dispensable [75]. On the other hand, indirect STING activation can be related to the induction of anti-inflammatory macrophages and aggravation of TB, as previously described [58]. Thus, STING can be paramount to the mechanism behind silicosis being a risk factor for TB and even other comorbidities. Supplementary to these investigations, *M. smegmatis* and *M. avium* ssp. *paratuberculosis* (MAP) were recently addressed on STING relevance. These non-tuberculous mycobacteria (NTM) differ in their virulence; while *M. smegmatis* is highly susceptible to macrophage killing, MAP can survive intracellularly, as other obligate pathogenic mycobacteria [79]. Despite both mycobacteria can induce STING activation, *M. smegmatis* induces high levels of type I IFNs that correlate with the clearance of the pathogen. Conversely, MAP produces a very weak activation of this pathway and can produce a persistent infection [79]. Interestingly, exogenous activation of type I IFN signaling reduced the MAP burden in vivo [79]. However, the definitive relevance of STING on controlling NTM infection is still missing. It is worth noting that, overall, the importance of STING in the mycobacterial infection context inversely correlates with the bug’s virulence [75,76,78,79]. Highly adapted pathogens can avoid STING recognition to promote infection, while less virulent mycobacteria activate several STING-dependent mechanisms. Additional studies are needed to confirm if this observation can be extended to other genera. The relevance of STING signaling in the infections discussed here is compiled in Table 1.

## 6. The Dichotomy of Type I IFN Responses: Host Resistance versus Susceptibility

Type I IFN signaling during bacterial insults, can have beneficial or detrimental outcomes for the host. The mechanisms are not completely understood and vary depending on the pathogen, experimental model, and the specific effector mechanisms linked to IFN signaling [80]. In the present study, we review the contrasting roles of type I IFN responses focusing on intracellular bacterial infections that engage the STING pathway.

### 6.1. Type I IFN-Inducing Protection in Bacterial Infection

Some of the earliest reports of type I IFN effects were demonstrated during chlamydial infections. In *Chlamydia trachomatis,* type I IFNs protected mice against infection [81,82], which was later correlated to its ability to deplete factors required for bacterial growth, such as the intracellular L-tryptophan [83].

Type I IFNs restricted *L. pneumophila* intracellular bacterial replication [84,85,86]. These protective effects correlated with inflammatory macrophage polarization and *Nos2* expression [85]. Further, in a distinctive mechanism, whereby type I IFNs can be beneficial to the host, it limited bacterial growth in *Legionella*-containing vacuoles inside macrophages. This pathway most likely involves the upregulation of antimicrobial ISGs [62]. Conversely, although type I IFN signaling was not required to limit *L. pneumophila* replication in mouse models of pulmonary infection [87,88], in association with IFN-γ, these cytokines mediated cell-autonomous resistance pathways that controlled *L. pneumophila* infection [62]. Additionally, type I IFN prevented cellular entry by the invasive gut bacteria, *S. flexneri* [89], through a pathway most likely associated with viperin (an evolutionary conserved IFN inducible protein). Viperin ectopic expression restricted bacterial entry and the loss of viperin enhanced intracellular bacterial levels [90]. The mechanism behind IFN, during *Burkholderia* infection, is particularly intriguing. Type I IFN signaling limited the replication of *Burkholderia cenocepacia* (an opportunistic pathogen generally associated with lung infections in patients with underlying immunodeficiencies), through the induction of a selective form of autophagy that facilitates cytosolic bacteria removal and prevents illness in immune-competent mice [91].

### 6.2. Detrimental Role of Type I IFN during Bacterial Infection

Several signaling pathways induced by IFNs impair anti-bacterial immunity, supporting bacterial pathogens. Although type I IFN signaling plays a protective role during *C. trachomatis* and *C. pneumoniae* infections [81,83], this cytokine is not universally protective against chlamydial species. The absence of IFNAR, rendered mice more resistant to *Chlamydia muridarum* in lung [92] and genital infections [93]. Resistance to infection and longer survival in IFNAR KO mice, correlated with reduced macrophagic apoptosis and apoptotic factors, including protein kinase R (PKR) and TNF-related apoptosis-inducing ligands (TRAILs). Interestingly, the depletion of lung macrophages dramatically increased *C. muridarum* replication, suggesting an important role of macrophages in clearing the infection [92].

The detrimental effect of type I IFN signaling in *L. monocytogenes*, was initially evidenced by enhanced resistance in IFNAR KO mice inoculated intravenously or intraperitoneally with *L. monocytogenes* [94,95,96,97]. Accordingly, disrupted IFN signaling during *L. monocytogenes*-induced enterocolitis, led to reduced bacterial loads at systemic sites [71]. The mechanisms described for these effects in *L. monocytogenes* infection are particularly diverse: resistance in IFNAR KO mice in *L. monocytogenes* infection was associated with decreased lymphocytes and macrophage apoptosis [97]; attenuated innate immunity, as a result of enhanced IL-10 secretion upon T cell apoptosis [95,98]; downregulation of IFN-γR (resulting in reduced protective IFN-γ signaling) [99]; and restriction of neutrophil recruitment [100,101].

In addition to the classical mechanisms described above, recent studies have demonstrated novel ways in which type I IFNs play detrimental roles during *Listeria* infection. The STING-dependent activation of type I IFN, correlated with *L. monocytogenes* pathology and inhibited cell-mediated immunity, a critical component for protection against intracellular pathogens [102]. Furthermore, Osborne et al. showed that type I IFN signaling is required for bacteria dissemination and efficient cell-to-cell spread. Using the cell surface-bound virulence protein (ActA), *L. monocytogenes* polarizes, driving bacterial motility in the cytosol. Type I IFN promoted polarization and the suppressed phagosome proteolysis of ActA, increasing actin-based motility and bacterial dissemination to perpetuate infection [103,104]. Recently, two bacterial products that facilitate *L. monocytogenes* infection, by directly inducing type I IFN signaling, were described. Firstly, Frantz et al. identified a small RNA, rli32, that induced higher IFN-β levels. The overproduction of rli32 promoted the intracellular survival of *L. monocytogenes*, and aided the resistance to hydrogen peroxide oxidative stress [105]. A second product, the RNA-binding protein Zea, also led to enhanced type I IFN signaling, and its inactivation decreased virulence [106]. The ISG ubiquitin-specific peptidase (USP18 or UBP43 (ubiquitin binding protein 43)) inhibits IFNAR signaling in a negative feedback loop by binding to IFNAR2 [107]. The participation of USP18, during *L. monocytogenes* infection, was recently described, and placed USP18 as a crucial element for the deleterious effect of type I IFN signaling. USP18 supported bacterial replication by inhibiting TNF-α antimicrobial effects [108]. However, the role of type I IFN during *L. monocytogenes* infection is still controversial, as it also plays a beneficial role during gastrointestinal infection [109] and plays no role in infection by the foodborne route [110]. These data reflect the myriad of mechanisms connected with IFN effects during bacterial injury and raise the interesting hypothesis that the infection route can be relevant to delineate the adverse or beneficial roles of type I IFN during infections.

*Francisella tularensis* is responsible for tularemia, a highly contagious and life-threatening respiratory disease. In macrophages, type I IFN had no role in the outcome of *F. tularensis* infection [111]. However, in mice, type I IFN was detrimental as it suppressed IL-17-associated recruitment of splenic neutrophils, a mechanism that, as in *Listeria* infections, was associated with impaired bacterial clearance and reduced survival [101,112,113]. *Francisella* recognition in macrophages relies on a coordinated STING/AIM2-dependent response [114]. Interestingly, IFNAR KO mice exhibited increased survival, while AIM2 KO mice showed increased susceptibility. Double KO (IFNAR/AIM2 KO) mice were protected against *F. novicida* infection, indicating that a type I IFN-mediated detrimental effect dominates the protective AIM2 responses. Several reports have demonstrated that IFNs can sensitize cells to apoptosis. This was also noticeable with *F. tularensis*, as the detrimental effects of type I IFN correlated with the induction of apoptotic caspases (e.g., caspases 3, 7, and 8) and apoptotic cell death [115].

Type I IFNs also play a detrimental role during other bacterial infections. In contrast to initial reports [89], host survival was enhanced in IFNAR KO and in IFN-β KO mice after infection with *S. typhimurium* [116,117]. Type I IFN signaling sensitized *S. Typhimurium*-infected macrophages to necrotic cell death, limiting pathogen control [117]. In addition to necroptosis, type I IFNs coordinate other pathways that are essential for mice survival following *Salmonella* infection, as IFN-β suppressed the expression of neutrophil chemokines and IL-1β/L-18 production [116]. Additionally, the absence of type I IFN signaling rendered mice more resistant to *Yersinia pestis* infection in a murine model of septicemic plague, and harbored augmented neutrophils, which protected mice from lethality [118,119]. *B. abortus* also benefits from the activation of type I IFN signaling. IFNAR KO mice were resistant to *B. abortus* infection, a phenotype associated with the elevated production of IFN-γ and NO, and reduced apoptosis compared to wild-type mice [120]. In a diverse fate, the induction of the UPR during *B. abortus* infection required type I IFN. Notwithstanding, treatment with exogenous IFN-β favored *B. abortus* survival in macrophages [49].

In the context of mycobacterial infections, the detrimental role of type I IFN signaling during TB has been demonstrated in both mice and humans [121]. In humans, an initial study using patients with active TB, showed a prominent type I IFN-inducible signature that correlated with disease severity [122]. Thenceforth, other studies in human TB confirmed these findings in patient cohorts from various geographic regions [123,124]. Accordingly, type I IFNs have been associated with *Mtb* virulence since type I IFNs were induced following infection with virulent *Mtb* strains, but not with nonvirulent strains [125,126,127]. Notwithstanding, the absence of type I IFN signaling decreased infection with highly virulent [125,126,128] and less virulent *Mtb* strains [129]. Robust evidence for the detrimental role of type I IFN during *Mtb* infection has been further established, as the induction of these cytokines exacerbated lung pathology and increased bacterial burden during *Mtb* infection in wild-type, but not IFNAR KO mice [125,130]. Similarly, the abrogation of negative regulators of type I IFN signaling, such as USP18, resulted in impaired *Mtb* clearance and decreased survival [131,132].

Although the cellular mechanisms linked to the detrimental effects of type I IFNs in *Mtb* infection remain unclear, diverse modes of action have been revealed. For instance, type I IFNs suppressed critical mediators of immunity against *Mtb,* namely IFN-γ and IL-1α/IL-1β, and promoted IL-10 production [125,133,134]. Furthermore, these cytokines modulated eicosanoid production [133] and the induction of IL-1Ra [135], both of which compromised protective IL-1 responses. Recently, resistance to *Mtb* infection was associated with the repression of type I IFN responses by the transcriptional regulator SP140 [136]. Strikingly, crosses to IFNAR KO mice to SP140 KO mice rescued the susceptibility to infection. Although the mechanisms associated with SP140 were not fully elucidated, these results suggest SP140 as a novel regulator of type I IFN induction that is crucial for resistance to bacterial infections [136]. Interestingly, the detrimental effects described here seem to be dependent on IFN-γ, since, in the absence of IFN-γ signaling, type I IFNs play a protective role during *Mtb* infection [137,138]. Further, type I IFN signaling is also detrimental in other mycobacterial strains. IFNAR KO mice infected with *M. smegmatis* showed enhanced survival [79]. Likewise, IFNAR blockage decreased mortality and bacterial numbers during *M. bovis* infection, and type I IFN inhibited the development of antimicrobial Th1 immunity and contributed to the progression of disseminated human leprosy, caused by *M. leprae* [139,140].

**Figure 2:**
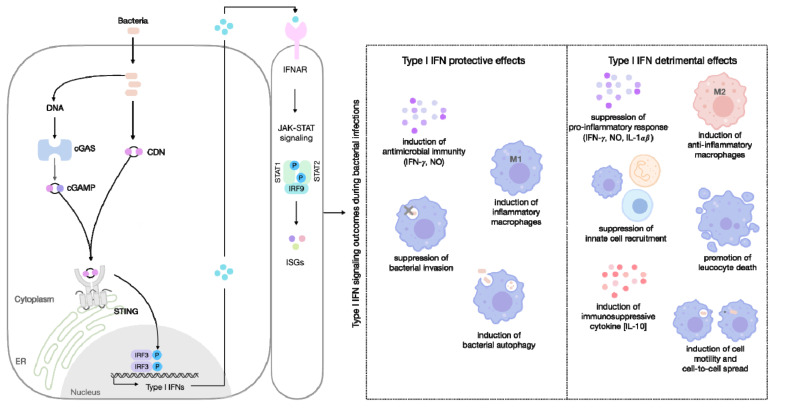
Type I IFN effects and mechanisms of action during bacterial infections. Stimulator of interferon genes (STING) direct sensing of bacterial cyclic di-nucleotides (CDN), or indirect sensing of bacterial DNA through cyclic GMP-AMP synthase (cGAS), leads to the induction of type I interferons (IFN). Type I IFNs bind to the type I IFN receptor (IFNAR), leading to Janus kinase (JAK)–signal transducer activator of transcription (STAT; JAK-STAT) signaling. Activation of JAKs, results in tyrosine phosphorylation of STAT1 and STAT2, leading to the formation of the IFN-stimulated gene factor 3 (ISGF3) (STAT1-STAT2-IFN-regulatory factor 9 (IRF9)) signaling complex. This canonical signal transducer complex, translocates to the nucleus and binds to IFN-stimulated response elements (ISREs) in gene promoters, leading to the induction of numerous IFN-stimulated genes (ISGs). Type I IFNs activate multiple components of host innate and adaptive immune responses, and type I IFN effects range from protective to detrimental to the host and include a variety of possible mechanisms of action. The main effects and mechanisms of action described for the bacteria addressed in this review are represented in the figure. cGAMP: cyclic GMP-AMP (cGAMP) ER: endoplasmic reticulum; IRF3: Interferon regulatory factor 3; IFN-γ: interferon gamma; NO: nitric oxide; M1: inflammatory macrophages; M2: anti-inflammatory macrophages; and IL: interleukin.

Many factors can influence the dual response related to type I IFN, and the reasons for the far-reaching role of these cytokines in bacterial infections remain incompletely understood. However, it appears that the ability of type I IFNs to both suppress and stimulate immune responses, is of critical relevance for the signaling outcome (Figure 2).

## 7. Concluding Remarks

A milestone concept of innate immunity is that host cells can detect bacteria and subsequently produce appropriate antibacterial responses. Bacterial genomic DNA and bacterial cyclic dinucleotides (CDNs) present in the cytosol are detected by cyclic GMP-AMP synthase (cGAS) and stimulator of interferon genes (STING), respectively. The mechanism and regulation of STING in inducing the expression of type I IFN have been extensively reported; however, the role of STING in protection against intracellular bacterial infections has been somehow controversial. Most of this opposite effect relies on the production of type I IFNs. Type I IFNs are mostly anti-viral, whereas interferon-stimulated genes (ISGs) induced by IFN-γ are predominantly important to control intracellular bacterial pathogens. For example, guanylate-binding proteins (GBPs) have antibacterial activities; in contrast, IL-10, alterations of eicosanoid production, and IL-1 receptor antagonist (IL-1RA) induced by type I IFN, impair anti-bacterial activity. More recently, Russell Vance’s group [136] dissected the mechanisms underlying the inappropriate type I IFN responses in bacterial infections, studying the super susceptibility to tuberculosis 1 (Sst1) locus in mice. They found that the *Sp140* gene within the *Sst1* locus represses type I IFN responses, and Sp140 KO mice were susceptible to *M. tuberculosis* and *L. pneumophila* infections. In contrast, an early study by Mancuso et al. [141] demonstrated that type I IFN is important for the protection against *Streptococcus penumoniae* and encapsulated *Escherichia coli* infections. A lack of type I IFN signaling was associated with the defective nature of IFN-γ, TNF-α, and NO production and susceptibility to infection.

Usually, bacterial DNA and CDNs are required to reach the host cell cytosol to activate the cGAS–STING pathway. As such, one would expect that the innate immune activities that occur in the host cell cytosol would be restricted to bacteria that damage or rupture phagosomal membranes. For example, the pathogenic bacterium *Listeria monocytogenes* escapes the phagosome and releases 3’3′-c-di-AMP into the cytosol to activate STING [9]. In addition, upon phagosomal rupture, some *Francisella novicida* bacteria are lysed within the cytosol, consequently releasing DNA for cGAS detection and STING pathway activation [113]. Furthermore, *L. pneumophila* mutants lacking the protein SdhA, and *S.* *typhimurium* mutants lacking the effector SifA, are unable to maintain the integrity of their phagosomes, resulting in the rupture and release of the bacterial components into the host cytosol for host receptor recognition [64,142]. Addressing how bacterial products are released in the cytoplasm of host cells will dramatically advance our understanding of cell-autonomous immunity to bacterial infections, and will reveal novel bacterial virulence strategies.

Finally, STING-dependent sensing of foreign nucleic acids predominantly enables the initiation of robust anti-pathogenic responses to protect the host. However, a better understanding of this pathway will provide significant insights into bacterial pathogenesis, and will open up avenues towards the development of innovative treatment strategies and vaccine designs.

## Figures and Tables

**Table 1:** The relevance of STING activation in bacterial infections addressed in this review.

Pathogen	Is STING Protective?	References
*Brucella* spp.	Yes	[49,55,56,74]
*Burkholderia* spp.	No	[67]
*Chlamydia* spp.	Inconclusive	[70]
*Legionella pneumophila*	Yes	[62,63,64]
*Listeria monocytogenes*	Yes (partially)	[65,71]
*M. avium* ssp.* paratuberculosis*	Inconclusive	[79]
*Mycobacterium smegmatis*	Inconclusive	[79]
*Mycobacterium tuberculosis*	No	[75]
*Salmonella typhimurium*	Yes (partially)	[72]
*Yersinia pestis*	Yes	[66]

## Data Availability

Not applicable.
